# Oxidative stress and altered lipid homeostasis in the programming of offspring fatty liver by maternal obesity

**DOI:** 10.1152/ajpregu.00049.2014

**Published:** 2014-04-30

**Authors:** Maria Z. Alfaradhi, Denise S. Fernandez-Twinn, Malgorzata S. Martin-Gronert, Barbara Musial, Abigail Fowden, Susan E. Ozanne

**Affiliations:** ^1^University of Cambridge Metabolic Research Laboratories, Wellcome Trust-MRC Institute of Metabolic Science, Addenbrooke's Hospital, Cambridge, United Kingdom; and; ^2^Department of Physiology, Development and Neuroscience, Centre for Trophoblast Research, University of Cambridge, Cambridge, United Kingdom

**Keywords:** developmental programming, nonalcoholic fatty liver disease, oxidative stress, metabolism, maternal obesity

## Abstract

Changes in the maternal nutritional environment during fetal development can influence offspring's metabolic risk in later life. Animal models have demonstrated that offspring of diet-induced obese dams develop metabolic complications, including nonalcoholic fatty liver disease. In this study we investigated the mechanisms in young offspring that lead to the development of nonalcoholic fatty liver disease (NAFLD). Female offspring of C57BL/6J dams fed either a control or obesogenic diet were studied at 8 wk of age. We investigated the roles of oxidative stress and lipid metabolism in contributing to fatty liver in offspring. There were no differences in body weight or adiposity at 8 wk of age; however, offspring of obese dams were hyperinsulinemic. Oxidative damage markers were significantly increased in their livers, with reduced levels of the antioxidant enzyme glutathione peroxidase-1. Mitochondrial complex I and II activities were elevated, while levels of mitochondrial cytochrome *c* were significantly reduced and glutamate dehydrogenase was significantly increased, suggesting mitochondrial dysfunction. Offspring of obese dams also had significantly greater hepatic lipid content, associated with increased levels of PPARγ and reduced triglyceride lipase. Liver glycogen and protein content were concomitantly reduced in offspring of obese dams. In conclusion, offspring of diet-induced obese dams have disrupted liver metabolism and develop NAFLD prior to any differences in body weight or body composition. Oxidative stress may play a mechanistic role in the progression of fatty liver in these offspring.

the prevalence of metabolic disorders has increased dramatically in the last few decades, particularly in developed and developing countries. Changes in lifestyle, diet, and physical activity—although contributing to this epidemic—do not explain the rapid increase in incidence. The importance of nutrient exposure before birth and postnatally in increasing the risk of metabolic disease in later life has been highlighted in a number of epidemiological studies (2, 15). With the number of overweight and obese women of child-bearing age increasing worldwide, this presents a worrying picture of a cyclical, transgenerational transmission of obesity. Models of maternal obesity have been developed to understand the mechanisms contributing to the programming of obesity in these offspring, which could result in intervention, treatment, or prevention.

The development of obesity often results in the onset of further metabolic complications, including insulin resistance and cardiovascular disease, together termed the metabolic syndrome. The hepatic manifestation of the metabolic syndrome (20), nonalcoholic fatty liver disease (NAFLD), is the most common liver disease in the Western hemisphere with prevalence as high as 30% (19). Features of metabolic syndrome in adult offspring, including NAFLD, have been observed in several animal models of maternal diet-induced obesity (4, 23, 28).

A number of pathogenic mechanisms for NAFLD have been suggested; however, a clear and consistent model of etiology has yet to be elucidated. Hepatic lipid deposition is predominantly attributed to dysregulated lipid homeostasis, which can result from increased fatty acid delivery from adipose tissue, increased synthesis of fatty acid via de novo lipogenesis, decreased mitochondrial β-oxidation, or a combination of these (25). The relative importance of each factor is likely to differ under different metabolic states (9, 10). NAFLD is also associated with insulin resistance, which is thought to be central to its pathogenesis (20). However, as obesity is a strong predictor of insulin resistance, it confounds the investigation of underlying mechanisms.

Oxidative stress has been suggested as a possible trigger for the onset of both insulin resistance and hepatic steatosis (8, 29). Studies have shown extensive association between oxidative stress markers and obesity, insulin resistance, and diabetes through interference of insulin signaling (13, 26, 39). Upregulation of pathways for reactive oxygen species (ROS) production and oxidative stress in insulin-responsive tissues (liver and adipose tissue) has been observed before the onset of insulin resistance (22), which could be suppressed by dietary restriction or antioxidant supplementation (5, 7, 16). Further, oxidative stress markers and inflammatory gene expression have been reported in the placenta of obese diabetic pregnancies (30), suggesting a role for oxidative stress in providing an abnormal developmental environment in offspring, which may lead to adverse metabolic effects.

In this study, we aimed to investigate the mechanisms underlying the onset of NAFLD in the offspring of diet-induced obese mice. To date, animal studies investigating the effects of maternal overnutrition on metabolic disease in offspring have studied offspring at 3 mo of age and older. By 3 mo of age, however, body weights have already diverged with offspring of obese dams being heavier and fatter than controls, thus confounding the investigation of underlying mechanisms (4, 24, 28). Therefore, we chose to study a time point prior to differences in offspring adiposity—8 wk of age—to investigate the etiology of hepatic steatosis in response to maternal diet-induced obesity. This gave us a better insight into uncoupling the interaction between multiple mechanistic factors contributing to the pathogenesis of NAFLD. Given the accumulating evidence of the importance of de novo lipogenesis in contributing to hepatic lipid deposition and the implications of oxidative stress in the onset of insulin resistance and hepatic steatosis in animal models, we also investigated these mechanisms in contributing to NAFLD onset in our nutritional programming model.

## MATERIALS AND METHODS

### 

#### Animal model.

All studies were approved by the local Ethics Committee and were conducted according to the Home Office Animals (Scientific Procedures) Act 1986. Primiparous female C57BL/6J mice were fed ad libitum either a standard chow RM1 diet [∼7% simple sugars, 3% fat, 50% polysaccharide, 15% protein (wt/wt), 10.74 kJ/g] or a semisynthetic energy-rich highly palatable obesogenic diet [∼10% simple sugars, 20% animal lard, 28% polysaccharide, 23% protein (wt/wt), 28.43 kJ/g] supplemented with sweetened condensed milk (Nestle, UK) (∼16% fat, 33% simple sugars, 15% protein, 13.7 kJ/g) and fortified with mineral and vitamin mix AIN93G. Both diets were purchased from Special Dietary Services (Witham, UK). After 6 wk on the diets, dams (*n* = 11 per group) were mated with chow-fed males for first pregnancy. The dams were allowed to litter, and the first litter was culled after weaning. This first pregnancy ensured the mice were proven breeders. After a week, mice were remated for a second pregnancy and *day 1* of pregnancy was defined by the appearance of a plug. Dams were maintained on their respective experimental diets throughout both pregnancies and lactation. Litter sizes were standardized to three males and three females on *postnatal day 3*. Offspring groups [control and maternal obesity (Mat-Ob)] were weaned onto standard chow (RM1) at 21 days of age for the remainder of the study. Body weight and food intake were recorded weekly. At 8 wk of age, following an overnight fast, offspring were killed by rising CO_2_ concentration. Tissues were dissected, weighed, snap frozen, and stored at −80°C until use. Female offspring were assessed in this study.

#### Dual-energy X-ray absorptiometry measurement of body composition.

Body composition analysis of offspring at 8 wk of age was performed by dual-energy X-ray absorptiometry (DEXA, Lunar PIXImus densitometer; GE Lunar, Madison, WI) immediately after killing.

#### Serum analysis.

Fed plasma glucose measurement was obtained from tail blood using a blood glucose analyzer (OneTouch Ultra, LifeScan) between 9 and 10 a.m., the day before the end of the study. All analysis on fasted serum was carried out following an overnight 16-h fast. Fasted insulin was measured using a Mercodia insulin ELISA kit (Mercodia AB, Uppsala, Sweden). Fasted triglycerides, free fatty acid (FFA), high-density lipoprotein (HDL), and cholesterol were measured using enzymatic assays (Dade Behring, Siemens Healthcare, Deerfield, IL). Low-density lipoprotein (LDL) was calculated from cholesterol, HDL and triglyceride levels using the formula LDL = cholesterol − HDL − (triglycerides/2.2).

#### Oxidative stress ELISA assays.

An 8-hydroxo-2-deoxy guanosine (8-OH-dG) EIA kit (StressMarq Biosciences no. SKT-120–96) was used to determine serum 8-OH-dG as a marker of DNA damage. Liver protein oxidation was measured using a 3-nitrotyrosine (3NT) ELISA kit (Abcam MitoSciences no. ab116691; Abcam, Cambridge, MA). Liver lipid peroxidation was measured using an OxiSelect 4-hydroxynonenal (4HNE) adduct ELISA kit (Cell Biolabs, no. STA-338). Mitochondrial complex I, II, and IV activities were measured using enzyme activity microassay kits (Abcam no. ab109721, no. ab109908, and no. ab109911, respectively) in isolated liver mitochondria (no. ab110170). All kits were used according to the manufacturers' protocols.

#### Western blot analysis.

The method has been described previously (35). In brief, liver protein lysates were standardized to a concentration of 2 mg/ml, and 40 μg total protein was resolved using SDS-PAGE. Table 1 lists the primary antibodies used in this study. Antibodies were diluted in 1× TBS, 0.1% Tween 20 containing either 1% nonfat dehydrated milk or 5% BSA. Horseradish peroxidase-conjugated secondary antibodies [anti-rabbit/mouse/goat antibody (Jackson ImmunoResearch, Stratech, Newmarket, UK)] were used. Protein expression was quantified densitometrically using AlphaEase software (AlphaInnotech, San Leandro, CA).

**Table 1.** Antibodies used in this study

Antibody	Source	Dilution	Company
5-Lipoxygenase	Rabbit	1:1000	Cell Signaling, no. 3289
Acetyl-CoA carboxylase	Rabbit	1:1000	Cell Signaling, no. 3676
Akt1	Mouse	1:1000	Cell Signaling, no. 2967
Akt2	Rabbit	1:5000	Cell Signaling, no. 2962
ATGL	Rabbit	1:1000	Cell Signaling, no. 2439
Catalase	Rabbit	1:10000	Abcam, no. ab1877
COX4	Rabbit	1:1000	Cell Signaling, no. 4844
CuZnSOD	Goat	1:20000	R&D Systems, no.AF3787
CYP4A14	Mouse	1:10000	Santa Cruz Biotechnology, no. sc-271983
Cytochrome *c*	Mouse	1:1000	Abcam, no. ab13575
eNOS	Rabbit	1:1000	Cell Signaling, no. 9572
Fatty acid synthase	Rabbit	1:10000	Cell Signaling, no. 3180
G6Pase	Goat	1:500	Santa Cruz Biotchnology, no. sc-27198
Glutamate dehydrogenase 1/2	Rabbit	1:1000	Cell Signaling, no. 9828
Glutathione reductase	Rabbit	1:1000	Abcam, no. ab16801
GPx1	Goat	1:2000	R&D Systems, no. AF3798
GSK3α/β	Mouse	1:200	Santa Cruz Biotechnology, no. sc-7291
Heme oxygenase 1	Rabbit	1:2000	Abcam, no. ab13243
iNOS	Rabbit	1:1000	Cell Signaling, no.2977
IRS1	Rabbit	1:1000	Upstate (Merck Millipore), no. 06-248
IRβ	Rabbit	1:200	Santa Cruz Biotechnology, no. sc-711
Lipoprotein lipase	Mouse	1:1000	Abcam, no. ab21356
MnSOD	Rabbit	1:10000	Upstate (Merck Millipore), no. 06-984
NOX4	Rabbit	1:500	Abcam no., no. ab60940
p110β	Rabbit	1:1000	Santa Cruz Biotechnology, no. sc-602
p47-phox	Goat	1:200	Santa Cruz Biotechnology, no. sc-23492
p85α	Rabbit	1:1000	Upstate (Merck Millipore), no. 06-195
PEPCK	Rabbit	1:1000	Santa Cruz Biotechnology, no. sc-32879
Phospho-Akt (Ser-437)	Rabbit	1:1000	Cell Signalling, no. 4058
Phospho-GSK3α/β (Ser-21/9)	Rabbit	1:1000	Cell Signaling, no. 9331
Phospho-IRS1 (Tyr-608)	Rabbit	1:1000	Upstate (Merck Millipore), no. 09-432
PKCζ	Rabbit	1:1000	Santa Cruz Biotechnology, no. sc-216
PPARα	Rabbit	1:200	Santa Cruz Biotechnology, no. sc-9000
PPARγ	Rabbit	1:1000	Cell Signaling, no. 2435
Pyruvate dehydrogenase	Rabbit	1:1000	Cell Signaling, no. 2784
SREBP1	Rabbit	1:200	Santa Cruz Biotechnology, no. sc-8984
TNFα	Rabbit	1:1000	Cell Signaling, no. 3707
Xanthine oxidase	Rabbit	1:200	Santa Cruz Biotechnology, no. sc-20991

ATGL, adipose triglyceride lipase; COX4, cytochrome-*c* oxidase complex IV; CuZnSOD, copper/zinc superoxide dismutase; CYP4A14, cytochrome *P*-450 4A14; eNOS, endothelial nitric oxide synthase; G6Pase, glucose-6 phosphatase; GPx1, glutathione peroxidase-1; GSK3, glycogen synthetase kinase; iNOS, inducible nitric oxide synthase; IRS1, insulin receptor substrate-1; IRβ, insulin receptor β; MnSOD, manganese superoxide dismutase; NOX4, NADPH oxidase 4; PEPCK, phosphoenolpyruvate carboxykinase; PPAR, peroxisome proliferator activator receptor; SREBP1, sterol regulatory element-binding protein 1.

#### Liver glycogen assay.

An amyloglucosidase assay was used to determine glycogen content of the liver (31). Frozen liver tissue (100 mg) was homogenized in 1 ml of ice-cold deionized water. Homogenate (100 μl) was incubated with 100 μl 50 mM acetate buffer (pH 4.5), with or without 70 units amyloglucosidase enzyme and 200 μl deionized water at 55°C for 10 min. 300 mM zinc sulfate (300 μl) and 300 mM barium hydroxide (300 μl) were added, and samples were mixed and centrifuged at 2,000 *g* for 10 min to deproteinize the samples. Supernatants were collected, and glucose concentrations were analyzed using a YSI 2300 STAT PLUS glucose analyzer (Yellow Springs Instruments Life Sciences, Yellow Springs, Ohio). Glycogen content relative to tissue weight was calculated as the glucose derived from enzymatic digestion by subtraction of free glucose (homogenate incubated without enzyme) from the total amount of glucose present in the liver homogenate (homogenate incubated with enzyme).

#### Liver lipid assay.

Liver lipids were extracted, according to a modified Folch method (12). Frozen liver tissue (100 mg) was thoroughly homogenized with 1 ml of a 2:1 ratio choloroform:methanol mixture (Sigma-Aldrich, Dorset, UK). Deionized water (200 μl) was then added to the mix, vortexed, and shaken for 10 min. Samples were centrifuged for 10 min at 16,100 *g* to generate a distinct organic and aqueous phase. The lower organic phase (500 μl) was collected into a preweighed glass tube and dried using a rotary evaporator for 2 h at 37°C, and the remaining lipid content was weighed.

#### Liver histology.

Liver sections were stained with Oil Red O for lipid content, according to a standard protocol. Briefly, liver fixed in 10% formalin were processed, paraffin-embedded, cut into 7-μm sections, and immersed in 30% sucrose solution overnight. Sections were rinsed in 60% isopropanol, immersed in freshly prepared Oil Red O solution for 15 min (Sigma-Aldrich, UK), rinsed again in 60% ispopropanol, and nuclei counterstained in Mayer's hemotoxylin. Images were captured on an inverted light microscope (Olympus BX41; Olympus, Southend-on-Sea, UK). Analysis was carried out using Adobe Photoshop Elements (Adobe Systems Software, Dublin, Ireland) on two fields of view from two sections per sample (*n* = 7 per group). Lipid droplets (red) were selected using the magic wand tool and quantified as a percentage of positively stained pixels in the field of view.

#### Statistical analysis.

Data were analyzed from one female offspring per litter; therefore, *n* refers to the number of litters per group. Data were analyzed by unpaired Student's *t*-test using Prism 5 (GraphPad, La Jolla, CA) unless stated otherwise. Protein expression data are presented as a percentage of mean expression of control group ± SE. All data are shown as means ± SE. For all data, *P* < 0.05 was considered statistically significant.

## RESULTS

### 

#### Offspring phenotype.

**Fig. 1.**
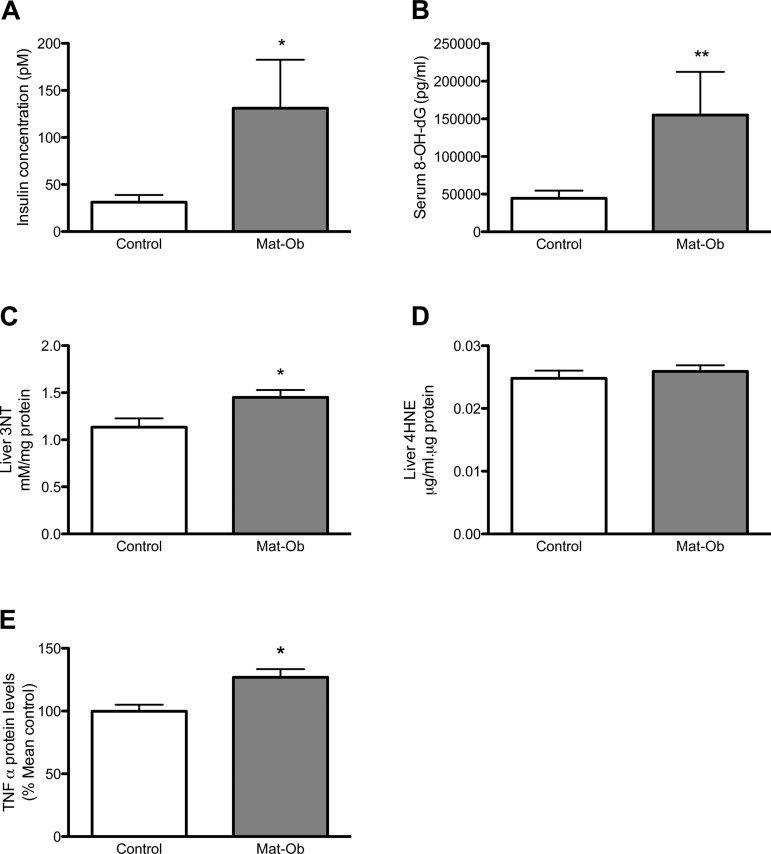
Insulin and oxidative stress markers. *A*: fasting serum insulin concentration. *B*: serum 8-hydroxy-2-deoxy guanosine (8-OH-dG) concentration. *C*: liver 3-nitrotyrosine (3NT) concentration. *D*: liver 4-hydroxynonenal (4HNE) concentration. *E*: protein levels of liver tumor necrosis factor-α (TNF-α). Data are presented as means ± SE for control (white bar) and maternal obesity (Mat-Ob) (gray bar) offspring. *n* = 6–8 per group. *P < 0.05, **P < 0.01.

Maternal diet did not affect offspring weight on *postnatal day 3* (Control 1.76 ± 0.06 g vs. Mat-Ob 1.63 ± 0.12 g, *n* = 11) or litter size (Control 7.6 ± 0.3 vs. Mat-Ob 6.0 ± 0.7, *n* = 11). At 8 wk of age, body weight, body composition, and liver weight were not significantly different between control and Mat-Ob offspring (Table 2). Cumulative food intake between weaning and *week 8* was similar between the two experimental groups (Control 107.6 ± 4.0 g vs. Mat-Ob 111.8 ± 3.4 g, *n* = 8 and *n* = 7, respectively). Levels of glucose, triglycerides, and FFA did not differ significantly between the two offspring groups at this age. Fasting total cholesterol and HDL cholesterol were significantly reduced in the Mat-Ob offspring compared with controls (*P* < 0.05) (Table 3). Fasting insulin levels, however, were significantly elevated in the Mat-Ob group compared with controls (Fig. 1*A*, *P* < 0.05).

**Table 2.** Body composition of 8-wk-old females

	Control	Mat-Ob
Total body weight, g	20.85 ± 0.98	19.16 ± 0.78
Fat mass, g	3.24 ± 0.17	3.20 ± 0.26
Lean mass, g	15.04 ± 0.12	14.77 ± 0.61
Bone mass density, g/cm^2^	0.04 ± 0.00	0.06 ± 0.02
% Fat mass	17.74 ± 0.63	17.83 ± 0.78
% Lean mass	83.03 ± 0.69	82.08 ± 0.68
Liver weight, g	1.12 ± 0.04	0.98 ± 0.06
% Liver weight	5.37 ± 0.04	4.90 ± 0.08

Data are presented as means ± SE; *n* = 6 per group. Mat-Ob, maternal obesity.

**Table 3.** Serum profile of 8-wk-old females

	Control	Mat-Ob
Total cholesterol, mmol/l	2.55 ± 0.12	1.93 ± 0.07*
HDL cholesterol, mmol/l	0.99 ± 0.04	0.85 ± 0.04*
LDL cholesterol, mmol/l	0.94 ± 0.07	0.76 ± 0.06
Triglycerides, mmol/l	0.75 ± 0.05	0.70 ± 0.11
FFA, mmol/l	1.03 ± 0.08	0.87 ± 0.05
Glucose (fed), mmol/l	9.5 ± 1.1	10.2 ± 1.1
Glucose (fasted), mmol/l	6.7 ± 0.3	5.9 ± 0.6

Data are presented as means ± SE; *n* = 6 per group,

* *P* < 0.05. HDL, high-density lipoprotein; LDL, low-density lipoprotein; FFA, free fatty acid.

#### Liver insulin signaling.

We investigated whether the hyperinsulinemia may be related to disrupted liver insulin signaling protein expression. However, no differences were detected in key hepatic insulin signaling molecules in the offspring in response to maternal obesity at 8 wk of age (Table 4).

**Table 4.** Insulin signaling protein expression in 8-wk-old female liver tissue

Expression, % mean control	Control	Mat-Ob
IRβ	100 ± 3	102 ± 4
IRS1	100 ± 15	120 ± 15
p-IRS1(Ser-307)	100 ± 16	109 ± 15
Akt1	100 ± 10	91 ± 11
Akt2	100 ± 4	99 ± 5
p-Akt(Ser-473)	100 ± 23	95 ± 30
p110β	100 ± 18	110 ± 11
p85α	100 ± 4	92 ± 7
PKCζ	100 ± 4	103 ± 5

Data are presented as means ± SE; *n* = 6 per group.

#### Oxidative stress damage.

Serum 8-OH-dG and liver 3NT, as markers of DNA and protein oxidative damage, respectively, were significantly increased in Mat-Ob offspring (Fig. 1, *B* and *C*, *P* < 0.01 and *P* < 0.05, respectively). Liver 4HNE, a marker of lipid oxidation, remained unchanged between the two experimental groups at this age (Fig. 1*D*). Liver TNF-α was significantly elevated in the Mat-Ob offspring compared with controls (Fig. 1*E*, *P* < 0.05).

#### Oxidative homeostasis.

**Fig. 2.**
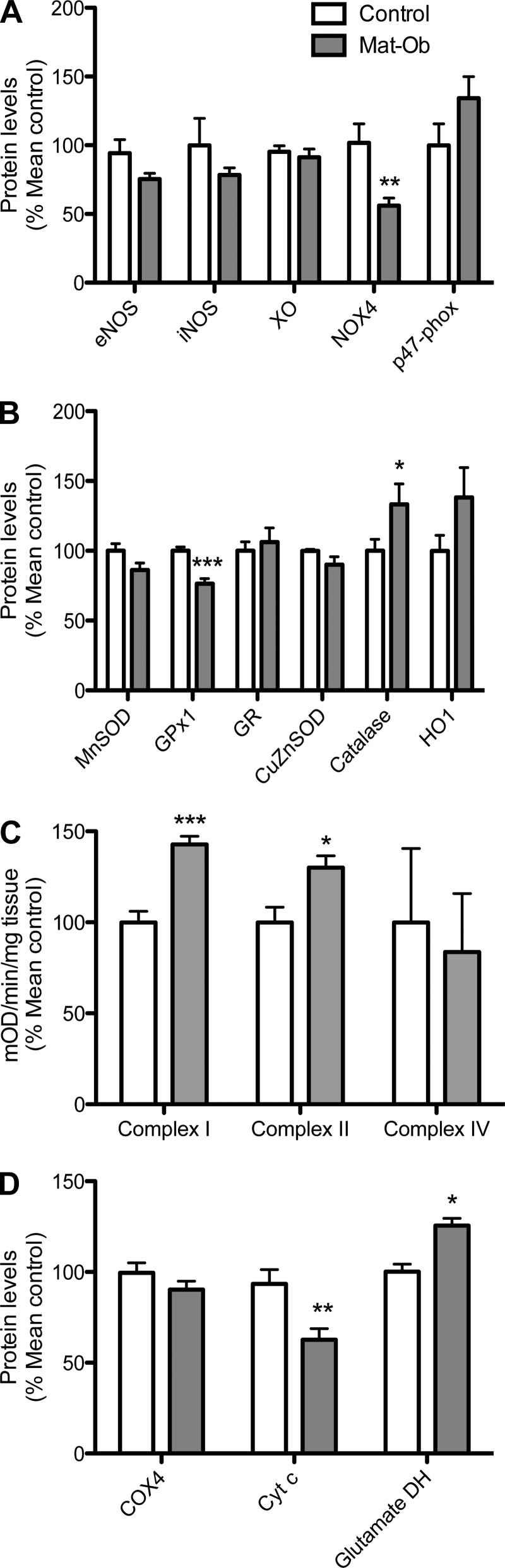
Oxidative homeostasis. *A*: protein levels of oxidative and nitrosative enzymes. eNOS, endothelial nitric oxide synthase; iNOS, inducible nitric oxide synthase; XO, xanthine oxidase; NOX4, NADPH oxidase 4. *B*: protein levels of antioxidant enzymes. MnSOD, manganese superoxide dismutase; GPx1, glutathione peroxidase-1; GR, glutathione reductase; CuZnSOD, copper/zinc superoxide dismutase; HO1, heme oxygenase-1. *C*: mitochondrial complex activities. *D*: protein levels of mitochondrial enzymes. COX4, cytochrome-*c* oxidase complex IV; Cyt *c*, cytochrome *c*; glutamate DH, glutamate dehydrogenase. Data are presented as means ± SE for control (white bar) and Mat-Ob (gray bar) offspring. *n* = 6–8 per group. *P < 0.05, **P < 0.01, ***P < 0.001.

Proteins involved in oxidative homeostasis were studied as possible contributors to increased oxidative stress. Livers of 8-wk-old Mat-Ob females had significantly lower NADPH oxidase 4 (NOX4) compared with controls (Fig. 2*A*, *P* < 0.01). There was also a significant reduction in the antioxidant enzyme glutathione peroxidase-1 (GPx1) (*P* < 0.001) and a significant increase in catalase levels (*P* < 0.05) in the Mat-Ob group compared with controls (Fig. 2*B*). No significant differences were observed in other proteins studied.

Uncoupling of mitochondrial oxidative respiration can also be a source of free radicals. Liver mitochondrial complex I and II activities were significantly increased in Mat-Ob offspring compared with controls (Fig. 2*C*, *P* < 0.001 and *P* < 0.05, respectively). Although complex IV expression and activities were not different, we observed a significant reduction in the expression of cytochrome *c*, the complex III-IV electron shuttle, in the offspring of diet-induced obese dams compared with controls (Fig. 2*D*, *P* < 0.01). Glutamate dehydrogenase (GDH), a marker of intact mitochondria, was elevated in this group (Fig. 2*D*, *P* < 0.05).

#### Liver lipid metabolism.

**Fig. 3.**
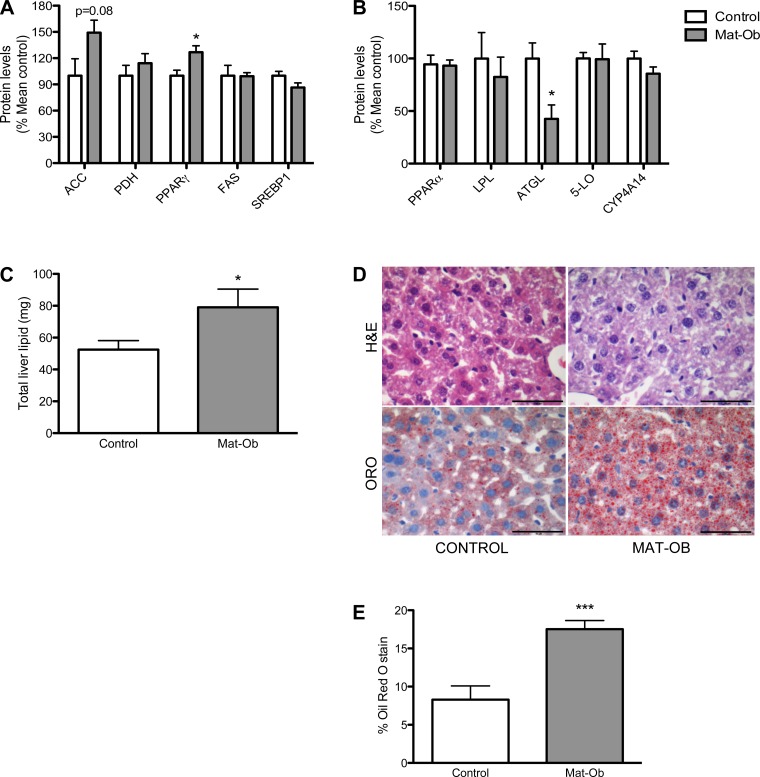
Liver lipid metabolism. *A*: protein levels of lipogenic enzymes. ACC, acetyl coA carboxylase; PDH, pyruvate dehydrogenase; PPARγ, peroxisome proliferator activator receptor γ; FAS, fatty acid synthase; SREBP1, sterol regulatory element-binding protein 1. *B*: protein levels of lipolytic enzymes. PPARα, peroxisome proliferator activator receptor α; LPL, lipoprotein lipase; ATGL, adipose triglyceride lipase; 5-LO, 5-lipoxygenase; CYP4A14, cytochrome *P*-450 4A14. *C*: total liver lipid content determined from 100 mg liver tissue. *D*: representative Oil-red-O (ORO) and hematoxylin-and-eosin (H&E) histology images. Scale bar is 50 μm. *E*: quantification of lipid droplet staining. Data are presented as means ± SE for control (white bar) and Mat-Ob (gray bar) offspring. *n* = 6–8 per group; *P < 0.05, ***P < 0.001.

We measured the levels of proteins involved in lipogenesis and lipolysis to identify possible changes in liver lipid homeostasis. PPARγ was significantly upregulated (*P* < 0.05), acetyl co-A carboxylase (ACC) protein levels were increased by 49% (*P* = 0.08) (Fig. 3*A*), and there was a significant reduction in liver triglyceride lipase (ATGL) in Mat-Ob offspring compared with controls (*P* < 0.05) (Fig. 3*B*). No significant differences were found in other proteins studied. These changes in lipid homeostasis were associated with increased total lipid content in Mat-Ob livers compared with the control group at 8 wk of age, as determined by both lipid assay and histological lipid content analysis (*P* < 0.05 and 0.001, respectively, Fig. 3, *C–E*).

#### Liver glycogen metabolism.

**Fig. 4.**
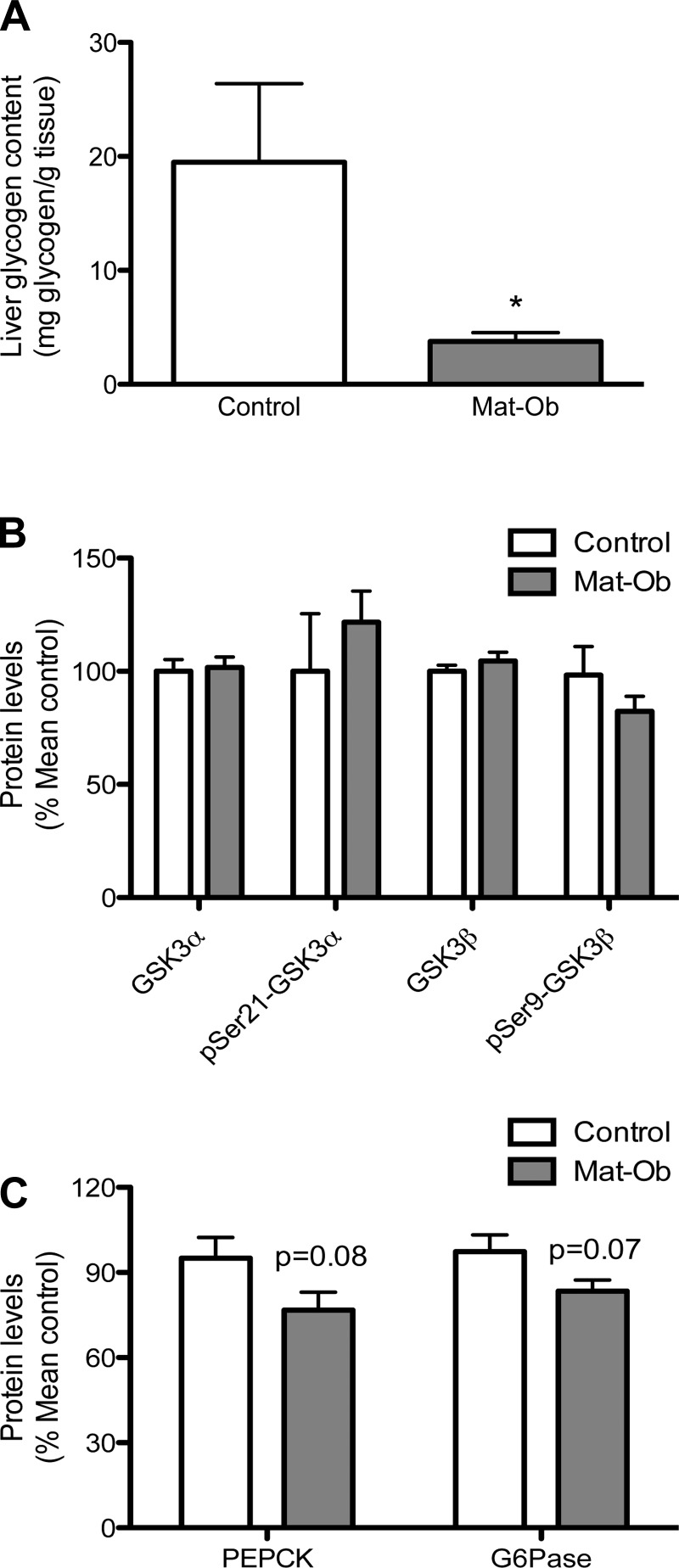
Liver glycogen metabolism. *A*: liver glycogen content. *B*: protein levels of total glycogen synthase kinase-3 isoforms and their phosphorylation levels. *C*: protein levels of gluconeogenic enzymes. PEPCK, phosphoenolpyruvate carboxykinase; G6Pase, glucose-6 phosphatase. Data are presented as means ± SE for control (white bar) and Mat-Ob (gray bar) offspring. *n* = 6–8 per group, *P < 0.05.

Total fasted liver glycogen content in the Mat-Ob group was significantly reduced compared with the control group (Fig. 4*A*, *P* < 0.05). Glycogen synthase kinase-3 (GSK-3) α and β protein levels were similar between the two experimental groups, and their phosphorylation levels were also not different (Fig. 4*B*). The levels of phosphoenolpyruvate carboxykinase (PEPCK) and glucose-6-phosphatase (G6Pase), key gluconeogenic enzymes, were reduced in expression in the Mat-Ob group compared with controls (Fig. 4*C*, *P* = 0.08 and 0.07, respectively).

#### Liver protein content.

There was a small, but statistically significant, reduction in total liver protein content in female Mat-Ob offspring compared with controls (Control 0.28 ± 0.01 vs. Mat-Ob 0.25 ± 0.00 mg protein/mg tissue, *n* = 6 and *n* = 5 respectively, *P* < 0.05).

## DISCUSSION

Obesity during pregnancy is a growing problem in both the developed and developing world. It is now evident that maternal obesity during pregnancy increases the risk of metabolic diseases in the offspring, including impaired glucose tolerance, insulin resistance, obesity, and NAFLD. The mechanisms underlying this programming are not well understood, and the close interrelationships between these pathologies make it difficult to dissect out primary metabolic defects. We rationalized, therefore, that to identify programming mechanisms for NAFLD, we would need to study offspring at an early time point, prior to the development of glucose intolerance and obesity.

We found that at 8 wk of age, there were no differences in body weight or adiposity between control and Mat-Ob offspring. Therefore, any metabolic differences can be attributed to differential programming of metabolic tissues. Hyperinsulinemia in Mat-Ob offspring before the onset of obesity indicate that they were insulin-resistant at this age. This is likely to be driven by insulin resistance in adipose tissue (11), as there were no differences in the hepatic expression of key insulin signaling proteins measured at this age, suggesting the liver is insulin-sensitive. The significance of reduced circulating cholesterol levels in Mat-Ob offspring is unclear. However, there is little evidence to suggest a link between serum cholesterol levels and hepatic fat deposition. One study has reported an association between hepatic steatosis and reduced HDL cholesterol levels in type 2 diabetic patients (37), whereas another study reported that the association was rendered nonsignificant after correction for insulin resistance (17), suggesting insulin resistance as a greater contributory factor to hepatic lipid deposition.

We suggest that hyperinsulinemia in Mat-Ob offspring, driven by peripheral insulin resistance, promotes the transcriptional upregulation of hepatic genes, including PPARγ, to promote de novo lipogenesis in the liver. Hormonal regulation of PPARγ activity has been previously demonstrated in cases where PPARγ activity was stimulated by insulin treatment (40, 41), and furthermore, its overexpression led to hepatic steatosis (42). As well as stimulating lipogenesis, insulin promotes lipid accumulation by inhibiting lipolysis. In the offspring of diet-induced obese dams, reduced expression of ATGL, the enzyme initiating the first step of lipid hydrolysis, is likely to contribute to hepatic lipid accumulation (14). Insulin has been reported to downregulate the transcription of ATGL via the mTOR pathway in mammalian adipocytes (6). Thus, the early development of peripheral insulin resistance in offspring of diet-induced obese dams may be a driver of hepatic triglyceride accumulation by regulating genes that are predominantly expressed in adipose tissue under normal conditions.

Hepatic insulin sensitivity in offspring exposed to maternal obesity declines by 12 wk of age, at which time the expression of key components of the insulin signaling pathway, including the insulin receptor and IRS1, is reduced (1, 21, 28); this could further exacerbate lipid accumulation and liver damage. Because we observed elevated markers of oxidative damage both in the circulation and in the liver, we investigated further whether oxidative dysregulation could contribute to NAFLD and the later development of insulin resistance; this confirmed an impaired hepatic antioxidant capacity through reduced GPx1 expression. Oxidative stress following HFD feeding has been observed to precede insulin resistance (22), the development of which can be prevented by antioxidant supplementation in rodents (5, 7, 16). Inflammation has been observed as early as the blastocyst stage in programming models (34). Thus, the raised hepatic TNF-α observed in this study provides further evidence for a state of enhanced oxidative stress. Increased oxidative damage markers 8-OH-dG and 4HNE have also been reported in livers of macaque fetuses exposed to a HFD in utero (23).

It is important to note that differences in animal models in terms of dietary composition, timing, and length of exposure confound the development of a single mechanistic model of programming. The majority of studies published to date investigating the hepatic effects of maternal overnutrition have utilized a HFD. In this study, dams were fed a high-fat, high-sugar diet to better reflect Western obesogenic environments. Differences in the metabolic responses of rodents fed a HFD vs. “cafeteria” Western diet have been previously noted, although both dietary challenges can induce obesity and hepatosteatosis, cafeteria-fed rats display increased inflammation in fat and liver tissue (32). Therefore, specificity in programming mechanisms from different dietary challenges cannot be dismissed and require further study. Nonetheless, our current findings are consistent with oxidative stress as an early mechanism through which offspring of diet-induced obese mothers are predisposed to NAFLD. The current findings suggest that the chronic oxidative damage resulting from maternal high-fat, high-sugar feeding is a programmed response independent of increased adiposity, and when coupled with hepatic lipid deposition, can contribute to insulin resistance and progressive hepatic steatosis and liver damage.

In our model of maternal diet-induced obesity, mitochondrial dysfunction is likely to be a source of ROS. While Bruce et al. (3) have previously reported reduced mitochondrial complex I, II/III, and IV activity in the livers of 15-wk-old offspring of HFD-fed dams, at 8 wk of age, we have observed here that Mat-Ob offspring complex I and II activities were increased along with reduced expression of the downstream electron carrier cytochrome *c*. This uncoupling of activity across the electron transport chain would likely result in increased electron leakage and free radical generation. Furthermore, expression of glutamate dehydrogenase—an enzyme abundantly expressed in liver mitochondria—was increased in Mat-Ob offspring. Serum levels of GDH are used as a marker of liver injury (27). The hepatic elevation in response to maternal diet-induced obesity may suggest compensatory mitochondrial proliferation in response to impaired function or hepatocyte necrosis (although the latter was not investigated in the present study). Thus, we hypothesize that mitochondrial dysfunction coupled with reduced antioxidant capacity contributes to the pro-oxidative environment at an early age. Catalase is an antioxidant enzyme known to be upregulated in response to oxidative stress (36). Therefore, the elevation of this protein in the Mat-Ob livers suggests a progressive compensatory response to ROS exposure. There is evidence to suggest a redox-centered pathogenic theory of NAFLD, where the redox state/ROS can modulate lipid metabolism, as well as insulin sensitivity (33). Furthermore, increased mitochondrial metabolism and complex III-generated ROS was found to induce PPARγ activity and adipocyte differentiation (38). The free radical biology of PPARγ in hepatocytes merits further investigation but suggests a further role for oxidative dysregulation in the underlying pathogenesis of NAFLD.

We observed reduced hepatic glycogen and protein in Mat-Ob offspring. This has also been observed in rats fed a HFD, where an inverse relationship between liver triglyceride and glycogen content was associated with insulin resistance (18), suggesting this is a compensatory response for the maintenance of hepatocyte integrity and function. Reduced lipolysis via ATGL and reduced gluconeogenesis via PEPCK and G6Pase suggests that glycogen is the predominant source of energy in the Mat-Ob offspring.

### Perspectives and Significance

In conclusion, our data provide evidence that exposure to an obesogenic environment during early developmental stages can prime offspring to increased susceptibility to nonalcoholic fatty liver disease. We propose a model in which the offspring of diet-induced obese dams develop early hyperinsulinemia from peripheral insulin resistance, thereby stimulating hepatic lipid deposition. Uncoupled mitochondrial respiration and oxidative stress in the liver further modulate lipid metabolism and contribute to the onset of hepatic insulin resistance, exacerbating hepatic steatosis with age. Hyperinsulinemia and reactive oxygen species may, therefore, present very early therapeutic targets for the prevention of NAFLD in susceptible individuals. This research emphasizes the importance of a balanced diet during pregnancy and lactation for the long-term health of subsequent generations.

## GRANTS

This research was supported in part by the Wellcome Trust (to M. Z. Alfaradhi), the Biotechnology and Biological Science Research Council (to D. S. Fernandez-Twinn: BB/F015364/1), the British Heart Foundation (to M. S. Martin-Gronert and S. E. Ozanne). S. E. Ozanne is a member of the University of Cambridge MRC Metabolic Diseases Unit. The research leading to these results has received funding from the European Union's Seventh Framework Programme (FP7/2007–2013), project Early Nutrition under grant agreement no. 289346.

## DISCLOSURES

No conflicts of interest, financial or otherwise, are declared by the authors.

## AUTHOR CONTRIBUTIONS

Author contributions: M.Z.A., D.S.F.-T., M.S.M.-G., and S.E.O. conception and design of research; M.Z.A., D.S.F.-T., and M.S.M.-G. performed experiments; M.Z.A., D.S.F.-T., and M.S.M.-G. analyzed data; M.Z.A., D.S.F.-T., B.M., A.L.F., and S.E.O. interpreted results of experiments; M.Z.A. prepared figures; M.Z.A. drafted manuscript; M.Z.A., D.S.F.-T., M.S.M.-G., B.M., A.L.F., and S.E.O. edited and revised manuscript; M.Z.A., D.S.F.-T., M.S.M.-G., B.M., A.L.F., and S.E.O. approved final version of manuscript.

## References

[1] Ashino NG Saito KN Souza FD Nakutz FS Roman EA Velloso LA Torsoni AS Torsoni MA Maternal high-fat feeding through pregnancy and lactation predisposes mouse offspring to molecular insulin resistance and fatty liver. J Nutr Biochem 23: 341–348, 2012 21543214 10.1016/j.jnutbio.2010.12.011

[2] Boney CM Verma A Tucker R Vohr BR Metabolic syndrome in childhood: association with birth weight, maternal obesity, and gestational diabetes mellitus. Pediatrics 115: e290–e296, 2005 15741354 10.1542/peds.2004-1808

[3] Bruce KD Cagampang FR Argenton M Zhang J Ethirajan PL Burdge GC Bateman AC Clough GF Poston L Hanson MA McConnell JM Byrne CD Maternal high-fat feeding primes steatohepatitis in adult mice offspring, involving mitochondrial dysfunction and altered lipogenesis gene expression. Hepatology 50: 1796–1808, 2009 19816994 10.1002/hep.23205

[4] Buckley AJ Keseru B Briody J Thompson M Ozanne SE Thompson CH Altered body composition and metabolism in the male offspring of high fat-fed rats. Metabolism 54: 500–507, 2005 15798958 10.1016/j.metabol.2004.11.003

[5] Campion J Milagro FI Fernandez D Martinez JA Diferential gene expression and adiposity reduction induced by ascorbic acid supplementation in a cafeteria model of obesity. J Physiol Biochem 62: 71–80, 2006 17217161 10.1007/BF03174068

[6] Chakrabarti P Kim JY Singh M Shin YK Kim J Kumbrink J Wu Y Lee MJ Kirsch KH Fried SK Kandror KV Insulin inhibits lipolysis in adipocytes via the evolutionarily conserved mTORC1-Egr1-ATGL-mediated pathway. Mol Cell Biol 33: 3659–3666, 2013 23858058 10.1128/MCB.01584-12 PMC3753874

[7] Dandona P Mohanty P Ghanim H Aljada A Browne R Hamouda W Prabhala A Afzal A Garg R The suppressive effect of dietary restriction and weight loss in the obese on the generation of reactive oxygen species by leukocytes, lipid peroxidation, and protein carbonylation. J Clin Endocrinol Metab 86: 355–362, 2001 11232024 10.1210/jcem.86.1.7150

[8] Day CP James OF Steatohepatitis: a tale of two “hits”? Gastroenterology 114: 842–845, 1998 9547102 10.1016/s0016-5085(98)70599-2

[9] Diraison F Beylot M Role of human liver lipogenesis and reesterification in triglycerides secretion and in FFA reesterification. Am J Physiol Endocrinol Metab 274: E321–E327, 1998 10.1152/ajpendo.1998.274.2.E321 9486165

[10] Diraison F Moulin P Beylot M Contribution of hepatic de novo lipogenesis and reesterification of plasma non esterified fatty acids to plasma triglyceride synthesis during non-alcoholic fatty liver disease. Diabetes Metab 29: 478–485, 2003 14631324 10.1016/s1262-3636(07)70061-7

[11] Fernandez-Twinn DS Alfaradhi MZ Martin-Gronert MS Duque-Guimaraes DE Piekarz A Ferland-McCollough D Bushell M Ozanne SE Downregulation of IRS-1 in adipose tissue of offspring of obese mice is programmed cell-autonomously through post-transcriptional mechanisms. Mol Metab 3: 325–333, 2014 24749062 10.1016/j.molmet.2014.01.007 PMC3986586

[12] Folch J Lees M Sloane Stanley GH A simple method for the isolation and purification of total lipids from animal tissues. J Biol Chem 226: 497–509, 1957 13428781

[13] Furukawa S Fujita T Shimabukuro M Iwaki M Yamada Y Nakajima Y Nakayama O Makishima M Matsuda M Shimomura I Increased oxidative stress in obesity and its impact on metabolic syndrome. J Clin Invest 114: 1752–1761, 2004 15599400 10.1172/JCI21625 PMC535065

[14] Haemmerle G Lass A Zimmermann R Gorkiewicz G Meyer C Rozman J Heldmaier G Maier R Theussl C Eder S Kratky D Wagner EF Klingenspor M Hoefler G Zechner R Defective lipolysis and altered energy metabolism in mice lacking adipose triglyceride lipase. Science 312: 734–737, 2006 16675698 10.1126/science.1123965

[15] Hochner H Friedlander Y Calderon-Margalit R Meiner V Sagy Y Avgil-Tsadok M Burger A Savitsky B Siscovick DS Manor O Associations of maternal prepregnancy body mass index and gestational weight gain with adult offspring cardiometabolic risk factors: the Jerusalem Perinatal Family Follow-up Study. Circulation 125: 1381–1389, 2012 22344037 10.1161/CIRCULATIONAHA.111.070060 PMC3332052

[16] Hotamisligil GS Arner P Caro JF Atkinson RL Spiegelman BM Increased adipose tissue expression of tumor necrosis factor-α in human obesity and insulin resistance. J Clin Invest 95: 2409–2415, 1995 7738205 10.1172/JCI117936 PMC295872

[17] Kantartzis K Rittig K Cegan A Machann J Schick F Balletshofer B Fritsche A Schleicher E Haring HU Stefan N Fatty liver is independently associated with alterations in circulating HDL2 and HDL3 subfractions. Diabetes Care 31: 366–368, 2008 18000185 10.2337/dc07-1558

[18] Kusunoki M Tsutsumi K Hara T Ogawa H Nakamura T Miyata T Sakakibara F Fukuzawa Y Suga T Kakumu S Nakaya Y Correlation between lipid and glycogen contents in liver and insulin resistance in high-fat-fed rats treated with the lipoprotein lipase activator NO-1886. Metabolism 51: 792–795, 2002 12037738 10.1053/meta.2002.32732

[19] Lazo M Clark JM The epidemiology of nonalcoholic fatty liver disease: a global perspective. Semin Liver Dis 28: 339–350, 2008 18956290 10.1055/s-0028-1091978

[20] Marchesini G Brizi M Bianchi G Tomassetti S Bugianesi E Lenzi M McCullough AJ Natale S Forlani G Melchionda N Nonalcoholic fatty liver disease: a feature of the metabolic syndrome. Diabetes 50: 1844–1850, 2001 11473047 10.2337/diabetes.50.8.1844

[21] Martin-Gronert MS Fernandez-Twinn DS Poston L Ozanne SE Altered hepatic insulin signalling in male offspring of obese mice. J Dev Origins Health Dis 1: 184–191, 2010 10.1017/S2040174410000231 25141786

[22] Matsuzawa-Nagata N Takamura T Ando H Nakamura S Kurita S Misu H Ota T Yokoyama M Honda M Miyamoto K Kaneko S Increased oxidative stress precedes the onset of high-fat diet-induced insulin resistance and obesity. Metabolism 57: 1071–1077, 2008 18640384 10.1016/j.metabol.2008.03.010

[23] McCurdy CE Bishop JM Williams SM Grayson BE Smith MS Friedman JE Grove KL Maternal high-fat diet triggers lipotoxicity in the fetal livers of nonhuman primates. J Clin Invest 119: 323–335, 2009 19147984 10.1172/JCI32661 PMC2631287

[24] Mouralidarane A Soeda J Visconti-Pugmire C Samuelsson AM Pombo J Maragkoudaki X Butt A Saraswati R Novelli M Fusai G Poston L Taylor PD Oben JA Maternal obesity programs offspring non-alcoholic fatty liver disease via innate immune dysfunction in mice. Hepatology 58: 128–138, 2013 23315950 10.1002/hep.26248

[25] Musso G Gambino R Cassader M Recent insights into hepatic lipid metabolism in non-alcoholic fatty liver disease (NAFLD). Prog Lipid Res 48: 1–26, 2009 18824034 10.1016/j.plipres.2008.08.001

[26] Nishikawa T Kukidome D Sonoda K Fujisawa K Matsuhisa T Motoshima H Matsumura T Araki E Impact of mitochondrial ROS production in the pathogenesis of insulin resistance. Diabetes Res Clin Pract 77 Suppl 1: S161–S164, 2007 17481767 10.1016/j.diabres.2007.01.071

[27] O'Brien PJ Slaughter MR Polley SR Kramer K Advantages of glutamate dehydrogenase as a blood biomarker of acute hepatic injury in rats. Lab Anim 36: 313–321, 2002 12144742 10.1258/002367702320162414

[28] Oben JA Mouralidarane A Samuelsson AM Matthews PJ Morgan ML McKee C Soeda J Fernandez-Twinn DS Martin-Gronert MS Ozanne SE Sigala B Novelli M Poston L Taylor PD Maternal obesity during pregnancy and lactation programs the development of offspring non-alcoholic fatty liver disease in mice. J Hepatol 52: 913–920, 2010 20413174 10.1016/j.jhep.2009.12.042

[29] Pessayre D Role of mitochondria in non-alcoholic fatty liver disease. J Gastroenterol Hepatol 22 Suppl 1: S20–S27, 2007 17567459 10.1111/j.1440-1746.2006.04640.x

[30] Radaelli T Varastehpour A Catalano P Hauguel-de Mouzon S Gestational diabetes induces placental genes for chronic stress and inflammatory pathways. Diabetes 52: 2951–2958, 2003 14633856 10.2337/diabetes.52.12.2951

[31] Roehrig KL Allred JB Direct enzymatic procedure for the determination of liver glycogen. Anal Biochem 58: 414–421, 1974 4827390 10.1016/0003-2697(74)90210-3

[32] Sampey BP Vanhoose AM Winfield HM Freemerman AJ Muehlbauer MJ Fueger PT Newgard CB Makowski L Cafeteria diet is a robust model of human metabolic syndrome with liver and adipose inflammation: comparison to high-fat diet. Obesity (Silver Spring) 19: 1109–1117, 2011 21331068 10.1038/oby.2011.18 PMC3130193

[33] Serviddio G Bellanti F Vendemiale G Free radical biology for medicine: learning from nonalcoholic fatty liver disease. Free Radic Biol Med 65C: 952–968, 2013 23994574 10.1016/j.freeradbiomed.2013.08.174

[34] Shankar K Zhong Y Kang P Lau F Blackburn ML Chen JR Borengasser SJ Ronis MJ Badger TM Maternal obesity promotes a proinflammatory signature in rat uterus and blastocyst. Endocrinology 152: 4158–4170, 2011 21862610 10.1210/en.2010-1078 PMC3199010

[35] Shelley P Martin-Gronert MS Rowlerson A Poston L Heales SJ Hargreaves IP McConnell JM Ozanne SE Fernandez-Twinn DS Altered skeletal muscle insulin signaling and mitochondrial complex II-III linked activity in adult offspring of obese mice. Am J Physiol Regul Integr Comp Physiol 297: R675–R681, 2009 19535678 10.1152/ajpregu.00146.2009 PMC2739782

[36] Sindhu RK Roberts CK Ehdaie A Zhan CD Vaziri ND Effects of aortic coarctation on aortic antioxidant enzymes and NADPH oxidase protein expression. Life Sci 76: 945–953, 2005 15589970 10.1016/j.lfs.2004.10.014

[37] Toledo FG Sniderman AD Kelley DE Influence of hepatic steatosis (fatty liver) on severity and composition of dyslipidemia in type 2 diabetes. Diabetes Care 29: 1845–1850, 2006 16873790 10.2337/dc06-0455

[38] Tormos KV Anso E Hamanaka RB Eisenbart J Joseph J Kalyanaraman B Chandel NS Mitochondrial complex III ROS regulate adipocyte differentiation. Cell Metab 14: 537–544, 2011 21982713 10.1016/j.cmet.2011.08.007 PMC3190168

[39] Urakawa H Katsuki A Sumida Y Gabazza EC Murashima S Morioka K Maruyama N Kitagawa N Tanaka T Hori Y Nakatani K Yano Y Adachi Y Oxidative stress is associated with adiposity and insulin resistance in men. J Clin Endocrinol Metab 88: 4673–4676, 2003 14557439 10.1210/jc.2003-030202

[40] Vidal-Puig A Jimenez-Linan M Lowell BB Hamann A Hu E Spiegelman B Flier JS Moller DE Regulation of PPAR gamma gene expression by nutrition and obesity in rodents. J Clin Invest 97: 2553–2561, 1996 8647948 10.1172/JCI118703 PMC507341

[41] Vidal-Puig AJ Considine RV Jimenez-Linan M Werman A Pories WJ Caro JF Flier JS Peroxisome proliferator-activated receptor gene expression in human tissues. Effects of obesity, weight loss, and regulation by insulin and glucocorticoids. J Clin Invest 99: 2416–2422, 1997 9153284 10.1172/JCI119424 PMC508081

[42] Yu S Matsusue K Kashireddy P Cao WQ Yeldandi V Yeldandi AV Rao MS Gonzalez FJ Reddy JK Adipocyte-specific gene expression and adipogenic steatosis in the mouse liver due to peroxisome proliferator-activated receptor gamma1 (PPARγ1) overexpression. J Biol Chem 278: 498–505, 2003 12401792 10.1074/jbc.M210062200

